# The influence of tourist visitation on the heterophyl to lymphocyte ratios and trophic values of Magellanic penguins (*Spheniscus magellanicus*) at Martillo Island, Argentina

**DOI:** 10.1093/conphys/coad063

**Published:** 2023-12-01

**Authors:** Sabrina Harris, Gabriela Scioscia, Andrea Raya Rey

**Affiliations:** Laboratorio de Ecología y Conservación de Vida silvestre, Centro Austral de Investigaciones Científicas, Consejo Nacional de Investigaciones Científicas y Técnicas, Houssay 200 (9410) Ushuaia, Tierra del Fuego, Argentina; Wildlife Conservation Society representación Argentina, Amenábar 1595 piso 2 oficina 19 (1426) CABA, Buenos Aires, Argentina; Laboratorio de Ecología y Conservación de Vida silvestre, Centro Austral de Investigaciones Científicas, Consejo Nacional de Investigaciones Científicas y Técnicas, Houssay 200 (9410) Ushuaia, Tierra del Fuego, Argentina; Laboratorio de Ecología y Conservación de Vida silvestre, Centro Austral de Investigaciones Científicas, Consejo Nacional de Investigaciones Científicas y Técnicas, Houssay 200 (9410) Ushuaia, Tierra del Fuego, Argentina; Wildlife Conservation Society representación Argentina, Amenábar 1595 piso 2 oficina 19 (1426) CABA, Buenos Aires, Argentina; Instituto de Ciencias Polares, Ambiente y Recursos Naturales (ICPA), Universidad de Tierra del Fuego (UNTDF), Walanika 250 (9410) Ushuaia, Tierra del Fuego, Argentina

**Keywords:** breeding, heterophil, lymphocyte, *Spheniscus magellanicus*, stable isotopes, stress, tourism

## Abstract

Wildlife tourism is increasing worldwide and monitoring the impact of tourism on wild populations is of the utmost importance for species conservation. The Magellanic penguin *Spheniscus magellanicus* colony at Martillo Island, Argentina, was studied in the 2016–2020 breeding seasons. In all seasons, adults and chicks belonged to: (i) an area close to or within the tourist trail or (ii) an area far from the tourist trail and out of sight of the tourists. Blood samples were taken for carbon and nitrogen stable isotope composition, in order to estimate trophic niches, and for smears that were made *in situ* and were then stained in the laboratory where leucocyte counts and differentiation were made under optical microscope. Heterophil to lymphocyte ratios were used as proxies of stress. Repeated sampling showed individual stress levels reduced while wintering. In 2017, stress levels and trophic values were lower than 2018 for the same individuals. Trophic levels did not differ between tourism and no tourism areas within each season, and differed between 2017 and the remaining seasons, indicating a possible diet shift that year. Stress levels were higher for the tourism area than the no tourism area for adults and chicks in all years except for 2020, when stress levels in the tourism area were lower and similar to the no tourism area that year and previous years. Vessel transit within the Beagle Channel and tourist visitation to the penguin colony was greatly reduced in 2020 due to the Covid-19 pandemic. A combination of internal characteristics and external factors may be affecting the stress physiology of individuals. Therefore, future research should include sampling of multiple aspects of penguin physiology, behaviour and environmental context in order to evaluate each effect on Magellanic penguin stress and, ultimately, inform the conservation of this iconic species in time.

## Teaser

Stress levels measured by heterophile to lymphocyte ratios of adult and chick Magellanic penguins *Spheniscus magellanicus* from Martillo Island were higher in areas exposed versus not exposed to tourism, except for 2020. Stress levels were also lower with lower trophic values for all individuals.

## Introduction

Wildlife tourism is increasing worldwide, and monitoring the human impact on wild populations is also gaining interest (Fennel, 2020). Tourism has become central to the economy of many places as thousands of dollars are spent in each season, fuelling cities’ and even countries’ economies as their main income (Schubert *et al.,* 2011; Romero *et al.,* 2021). Ecotourism can promote awareness of the fragility of wild places, the human–wildlife connectivity and the importance of species conservation (Müller, 1999; Wearing and Neil, 2009). On the downside, human presence can also have detrimental effects on breeding populations either through direct interference causing behaviour and stress changes and pathogen transmission (Stoddard *et al.,* 2009; Geffroy *et al.,* 2017) or indirectly by changing prey distribution and abundance, introduction of invasive species and harming habitat with pollutants including microplastics (Hilton and Cuthbert, 2010; Susanti *et al.,* 2020). Unregulated human visitation to seabird colonies has had catastrophic effects on many seabird populations in the past (Coker, 1908; Anderson and Keith, 1980; Martin *et al.,* 2014), therefore monitoring behaviour, diet, health, stress and breeding parameters of seabird populations exposed to tourism is paramount for sustainability of ecotourism and seabird populations over time (Hofer and East, 1998; Ellenberg, 2017).

Breeding is one of the most energetically stressful moments of seabird life cycles. The physiological cost of egg production and chick rearing pushes individuals to a state of increased functional strain particularly susceptible to external stressors. Breeding individuals exposed to stressors such as prey depletion, human presence, contaminants, parasite exposure or heat stress, among others, at this fragile time could affect their capacity to incubate eggs and raise chicks and, ultimately, force them to abandon the breeding event (Boersma *et al.,* 2013; Trathan *et al.*, 2014). Increased stress levels have been detected in seabirds in years of low food availability (Kitaysky *et al.,* 2007), exposure to contaminants (Costantini *et al.,* 2014), linked to shifts in diet (Barger and Kitaysky, 2011) or to heat stress due to increased temperatures (Oswald *et al.,* 2008; Oswald and Arnold, 2012). Moreover, stress levels vary given different stages of the breeding season (Vleck *et al.,* 2000; Dehnhard *et al.,* 2011; Colominas-Ciuró *et al.,* 2022a) and for different locations within the colony (Viblanc *et al.,* 2014). In addition, at colonies exposed to tourism, differences in stress levels have been detected in areas with vs. without human visitation (Walker *et al.,* 2008; Villanueva *et al.,* 2011; Barbosa *et al.*, 2013; Palacios *et al.,* 2018).

The avian immune system is divided into innate and acquired immunity (Rose, 1979; Kaspers *et al.,* 2021). Heterophils play a vital role in the innate response, as they are macrophagic cells with action against pathogenic microbes and also increase under mildly or moderately stressful situations. The acquired response involves lymphocytes, both cells-mediated and through secretion of antibodies (Rose, 1979). Lymphocytes are also reduced under chronic stress as a consequence of a sustained increased level of glucocorticoids (Taves *et al.*, 2017; Brooks *et al.,* 2022). Therefore, the increase in heterophil to lymphocyte (H/L) ratio can be used to detect the presence of sustained physiological stress (Siegel, 1980; Maxwell and Robertson, 1998; Davies *et al.,* 2008). Monitoring the H/L of the same individuals throughout and even between breeding seasons can be used to estimate the physiological cost of each breeding stage and potential interannual changes in stress of the individuals. In addition, the long-term monitoring of the H/L of a breeding population may provide insights as to the potential effects of changes in diet, weather or the impact tourism may have on the health of the population in time (Kitaysky *et al.,* 2007; Graña Grilli *et al.,* 2018; Whitehead and Dunphy, 2022). Eosinophils have a less clear immune function but seem to be linked to allergic reactions and parasite infections (Maxwell, 1987). Eosinophil to lymphocyte (E/L) ratio can also be estimated in order to determine if there are changes in other immune functions such as inflammatory responses due to parasites (Clarke and Kerry, 1993).

Physiological stress due to shifts in diet or nutritional deficiencies has also been described in many species (Jodice *et al.,* 2006; Colominas-Ciuró *et al.,* 2022b). In seabirds, years of lower prey availability have been linked to higher stress levels and lower breeding performance (Kitaysky *et al.,* 2007; Will *et al.,* 2015; Fromant *et al.,* 2021). Seabird diet can be inferred through stable isotope analysis such as blood tissue δ^15^N and δ^13^C (Inger and Bearhop, 2008). By monitoring the diet of seabirds over time, transient or permanent shifts in prey availability can be detected which, in turn, are expected to impact stress levels of seabirds (Thompson and Hamer, 2000). Higher trophic level prey such as fish and squid may require more skills to capture and therefore entail higher effort which may, in turn, translate into higher stress of the predator (Will *et al.,* 2015; Tate *et al.,* 2021). Stress inferred by H/L and trophic levels can be assessed for the same individuals in order to determine if certain levels of stress coincide with particular trophic signatures that may be reflecting differences in foraging costs or the impact of the nutritional value of prey on seabird physiology. In addition, extreme environmental conditions such as heatwaves have detrimental effects on seabirds, affecting breeding success and even survival of adults (Cook *et al.,* 2020; Holt and Boersma, 2022; Olin *et al.,* 2023). Less extreme environmental conditions may have sublethal effects on individuals who may suffer a strain on their physiology, which may translate into increased stress linked to thermoregulation (Tate *et al.,* 2021). Therefore, evaluating the environmental conditions individuals endure is also important to have a more complete understanding of their physiology and behavior (Oswald and Arnold, 2012).

Magellanic penguins (*Spheniscus magellanicus*) inhabit the coasts of southern South America, from 42°S down to the Beagle Channel, and including the Malvinas (Falkland) Islands (Boersma *et al.,* 2013). This species has been studied in many aspects including impact of tourism on stress levels (measured in corticosterone and in H/L ratios) in the northern region of Argentina at Punta Tombo and San Lorenzo colonies. Differences have been detected in behavioural and glucocorticoid stress hormone patterns in adults and chicks between locations at Punta Tombo being higher in areas with vs. without tourist visitation (Walker *et al.,* 2005a, 2008) and no differences between zones at San Lorenzo colony, with more recent and less tourism (Palacios *et al.,* 2018). Chicks also showed acute stress (as measured by increases in glucocorticoid stress hormones after capture restraint) in tourist-visited areas as compared to non-visited, but only during the days immediately after hatching (Walker *et al.,* 2005a). Magellanic penguins also coexist with numerous endo- and ectoparasites (Sallaberry-Pincheira *et al.,* 2015; Prichula *et al.,* 2020; Uhart *et al.,* 2020) which activate immune responses particularly elevating heterophils (which have macrophagic action on pathogenic microbes such as *Salmonella* sp. *E. coli*) and eosinophils in parasitic infections (endoparasites such as intestinal nematodes (Campbell and Ellis, 2007)).

During the breeding season, seabirds are limited in the range they can cover in search of food and must rely on prey close to the colony so they do not fully digest the food in their stomachs before returning and have food for their chicks (less than 50 km in the case of Magellanic penguins from Martillo Island (Harris *et al.,* 2020)). The most abundant prey in the Beagle Channel and available for Magellanic penguins are squat lobster *Munida gregaria* and Fuegian sprat *Sprattus fuegensis* (Scioscia *et al.,* 2014). Fuegian sprat has cyclical movements and therefore its availability depends on the time of year and the time of day (Diez *et al.,* 2018). During November–December sprat enters the channel and becomes an increasingly preferred prey, as it is more digestible and therefore offers higher nutritional content for the growing chicks (Thompson, 1993). However, shifts in abundance may occur some years and diet of penguins also adjusts (Scioscia *et al.,* 2014). These changes in diet may be linked to higher or lower stress levels as foraging effort may increase if prey is scarce or the nutritional value of poorer quality diet may have a cost on physiology (Graña Grilli *et al.,* 2018). Increased or decreased stress levels may ultimately impact breeding performance (Moreno *et al.,* 2001; Wanless *et al.,* 2005; Satterwaite *et al.,* 2012; Barrionuevo *et al.,* 2018).

Martillo Island is one of several islands in the Beagle Channel and is home to 3500 breeding pairs of Magellanic penguins (Raya Rey *et al.,* 2014 and unpublished data). This colony was founded in the 1970s and has had sustained tourist visitation since the 1980s, but people only began disembarking and thus being in direct interaction with penguins in 2004. Tourism is regulated under the Onashaga Commitment whereby the places tourists can visit are delimited by a trail, and the total number of tourists at a given moment on the island is limited to 20 (a maximum of 120 per day during 5 or 6 one-hour visits). In contrast, more than 29 000 tourists visit them in a given season on board vessels (Raya Rey unpublished data, Schiavini and Yorio, 1995). It is of interest to determine if stress levels detected in penguins differ between areas with vs. without human visitation in normal years and without tourism (2020 season). In addition, it is of interest to determine whether differences in diet measured by stable isotope composition in blood could be influencing baseline stress levels of penguins over the years. Stress levels measured by H/L ratios of adult and chick Magellanic penguins exposed to tourism are expected to be higher than those not exposed to tourists. In addition, individuals with higher trophic levels are expected to have higher stress levels than individuals with lower trophic levels, assuming increased foraging effort on higher tropic level prey has a negative impact on stress.

## Methods

**Figure 1:**
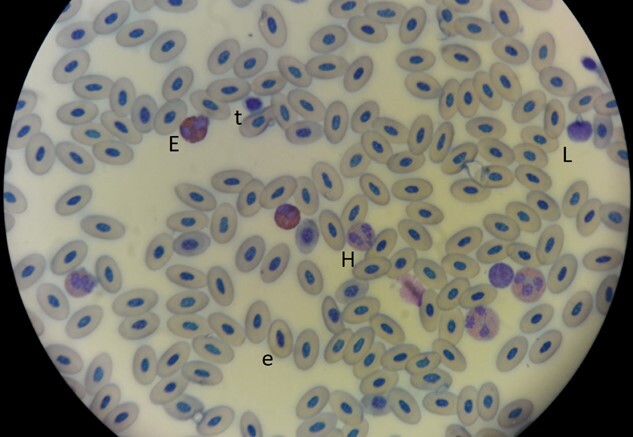
Blood smear of Magellanic penguin with the following cell types: (H) heterophil, (L) lymphocyte, (E) eosinophil, (e) erithrocite, (t) thrombocite stained with Giemsa (1:7) at 100$\times$ magnification (immersion Oil).

Research took place at Martillo Island, Beagle Channel, Argentina, where adult and chick Magellanic penguins were sampled in five breeding seasons: 2016–2020 (Table 1). Breeding seasons begin in September and end in March the following year; therefore, from now on the seasons will be named by the year the season began. Two nesting areas were defined: the tourist area (within or less than 10 m from the tourist trail) and the no tourist area (more than 500 m away and not in sight from the tourist trail). In all cases, individuals were captured at their nest using a hook, weighed using a Pesola macro-line hanging spring scale (10 kg, 100 g precision, Pesola, Switzerland), their beaks were measured using a dial caliper (0.02 mm precision following Gandini *et al.,* 1992) and three drops of blood were extracted from the tarsal vein, one was preserved in alcohol 70% and another two were placed on microscope slides and smears were made in the field (duplicates). In the lab, smears were fixed with alcohol 70% and dyed with Giemsa stain (diluted 1 in 7 with distilled water) for 15 min. Dyed smears were observed under optical microscope at 1000$\times$ with oil immersion and white blood cells were identified and counted (heterophyls, lymphociets, eosinophils, basophils and monocites). Smears were used only if more than 100 leucocytes were identified per slide in order to sample a representative proportion of cell types per individual. A total of 20% of smears had to be discarded due to poor quality. The stress estimation given by cell counts such as in the present work is not affected by short-term acute stress such as handling stress (Vleck *et al.,* 2000). However, all handling times were under 4 minutes in order to minimize stress to penguins caused by our manipulation. The percentage of heterophils, lymphocytes and eosinophils as well as (H/L) and (E/L) were estimated by the same observer, S.H. (following Campbell, 1995; Fig. 1). Ratios were log 10 transformed in order to obtain normality of ratios (following Minias, 2019), normality of the log transformed data was verified with a Fisher test. Sex of adults was determined by the relation between width and length of the beak following Scioscia *et al.* (2016).

**Table 1:** number of adult and chick Magellanic penguins *Spheniscus magellanicus* sampled for smears in each zone (no tourism (n), tourism (t)), stage: late (March), early (September), middle (December, January) and season (from September to March the following year). Individuals resampled the following stage indicated with *

		**2016**	**2017**		**2018**	**2019**	**2020**
		**Late**	**Early**	**Middle**	**Middle**	**Middle**	**Middle**
**Adults**	**n**	15*	15*	7*	4* + 2	10	15
	**t**		7*	7*	6* + 1	16	15
**Chicks**	**n**				13	6	16
	**t**				9	8	14

### Stress levels within and between breeding seasons

**Figure 2:**
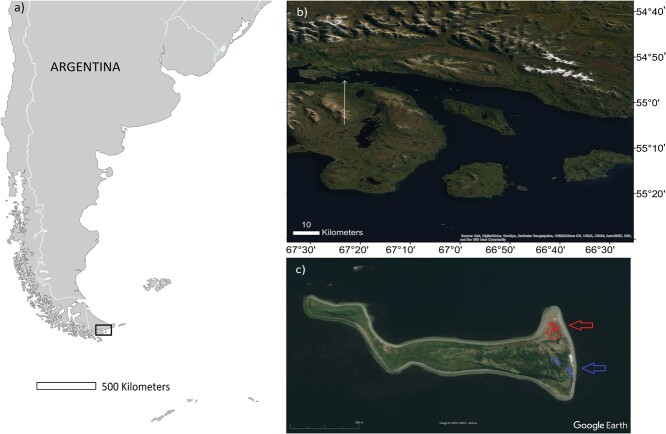
**a**) Location of the breeding colony in Argentina. **b**) Enlarged sector from **a**) with Martillo Island within the Beagle Channel indicated with arrow. **c**) Martillo Island, tourist trail (filled area) and approximate tourism area circled with a line in the top right sector of the island and approximate no tourism areas in the middle-right sector of the island.

In 2016, 2017 and 2018 a first exploratory study was done to determine if there were changes in (H/L) and (E/L) ratios and weights of adults throughout the breeding season and between breeding seasons. Penguins were identified by the code of the previously inserted subcutaneous chips (Rumitag SL, Barcelona, Spain). A group of the identified penguins that belonged to an area not exposed to tourism were sampled after moult in March 2017 (late 2016 season) and then again before laying in September 2017 (early 2017 season). In addition, a group of the birds from the area not exposed to tourism and a group exposed to tourism (nesting within 50 m from and in sight of the tourist trail, Fig. 2) were resampled during chick rearing that same season (from now on middle season, in December 2017–January 2018) and again during chick rearing (middle) of the following season (in December 2018–January 2019, Table 1). Individuals were also weighed and measured. In all cases, generalized linear mixed effects models (GLMM) were run with the log_10_ transformed H/L or E/L as a function of stage (late 2016 to early 2017, early 2017 to middle 2017 or middle 2017 to middle 2018) with sex as fixed factor and bird identity as a random effect.

### Across the years

In 2018, 2019 and 2020 breeding seasons, sampling took place in the previously defined tourism and no tourism areas. During chick rearing (middle) of 2018 season (between mid-December 2018 and the beginning of January 2019), data were obtained from tourism and no tourism adults (including the adults resampled from 2017) and tourism and no tourism chicks. In the middle of the 2019 season and the middle of 2020 season (January 2021), data was collected from tourism and no tourism adults and chicks. In all cases adults were sampled when chicks were less than 20 days old and chicks were sampled when they were less than 40 days old. Individuals were then monitored until the end of the breeding season to ensure breeding was not affected. In 2019 and 2020 seasons, care was taken to sample individuals belonging to different sectors of each area in different years in order not to resample individuals in successive seasons. Penguins have high nest site fidelity; therefore, by choosing different sectors of the tourism and no tourism areas each year, the chances of resampling individuals were minimized. Generalized linear models were run for the log_10_ transformed H/L or E/L with sex, year and area (tourism and no tourism) as fixed affects. Data was tested for normality with Shapiro–Wilk’s test and homoscedasticity with the residuals vs. fitted plot. All models were run with (lme) package in R (R Core Team, 2020). Significance was set at *P* < 0.05.

### Trophic values

In order to determine the trophic niche of each group of individuals and compare amongst areas and years, the remaining sets of blood samples preserved in 70% alcohol were dried in an oven at 50°C for 48 h and weighed into tin capsules. Dry samples were sent to Laboratorio de Isótopos Estables en Ciencias Ambientales (LIECA, Mendoza, Argentina) for carbon and nitrogen stable isotope composition determination via a Thermo Scientific DELTA V Advantage spectrometer coupled via an interface ConFlo IV to an Elemental Flash 2000 analyser (Thermo, Massachusetts, USA). Sample precision based on repeated sample and reference material was 0.1‰ for δ^13^C and δ^15^N. Stable isotope values are expressed in δ notation in per mil units (‰), according to the equation:

δX = [(R_sample_/R_standard_)-1]x1000

Carbon isotopic values were corrected for the Suess effect (Keeling 19 679) using the following formula:

δ^13^Ccorr = δ^13^C—((2020-year)*0.002).

Carbon and nitrogen isotopic values were compared using GLS (general least squares models) with year and area (with or without tourism) as fixed effects. Biplots were made and overlap of areas of ellipses estimated as a percentage overlap of the total summed ellipse areas corrected for small sample size (SEAc) between pairs of groups of individuals were estimated using SIBER package (Jackson *et al.,* 2011) in R.

### Natural and anthropogenic factors in 2017–2020

Weekly mean sea surface temperature (SST) for the area surrounding the colony (within latitudes 55.25°S and 54.75°S and longitudes 67.25°W and 66.75°W) was obtained from the Climate Change Initiative—European Space Agency https://climate.esa.int/es/ (date last accessed 18 March 2023) web page and monthly mean and standard deviation (SD) were calculated. Ambient temperature (mean, maximum and minimum daily values) and daily rainfall for 2017–2020 were obtained from the Servicio de Información Ambiental y Geográfico https://cadic.conicet.gov.ar/informacion-meteorologica/ (date last accessed 20 April 2023) for Ushuaia city (lat 54.8°S; long 68.3°W) and mean weekly values were estimated for each variable. Information on cruise ship movements within the Beagle Channel for 2017–2020 was obtained from the INFUETUR web page https://infuetur.gob.ar/estadistica/temporada_cruceros (date last accessed 2 May 2023), and information on total vessel transit was obtained from the Dirección provincial de puertos de Ushuaia web page https://www.dpp.gob.ar/web/puerto-ushuaia/estadisticas/evolucion-de-buques/ (date last accessed 20 April 2023). Mean monthly values of SST, ambient temperature and rainfall were compared amongst months and years with a F test and amongst the studied seasons with a *t* test. Significance was set at *P* < 0.05.

## Results

### Stress levels within and between breeding seasons

**Figure 3:**
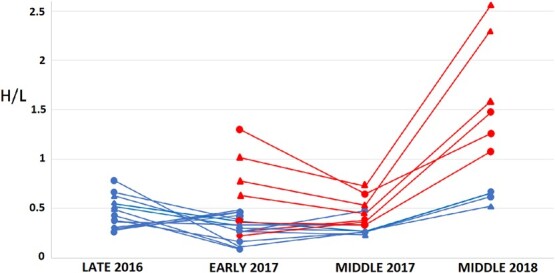
Stress levels estimated by Heterophil vs. lymphocyte (H/L) of the same adult Magellanic penguins during late 2016 (pre moult in March 2017), early 2017 (pre-laying in September 2017), middle 2017 (chick rearing in December 2017–January 2018) and middle 2018 (chick rearing in December 2018–January 2019). No tourism area (with lower H/L values) and tourism area (with higher H/L values), triangles for males and circles for females.

**Figure 4:**
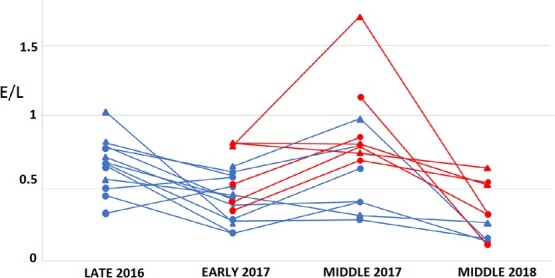
Eosinophil vs. lymphocyte (E/L) of the same adult Magellanic penguins during late 2016 (pre moult in March 2017), early 2017 (pre-laying in September 2017), middle 2017 (chick rearing in December 2017–January 2018) and middle 2018 (chick rearing in December 2018–January 2019). No tourism area (with lower E/L values) and tourism area (with higher E/L values), triangles for males and circles for females.

Breeding adult Magellanic penguins from the no tourism area had higher H/L ratios after moult at the end of the 2016 season (0.5 ± 0.2), than before laying the following season in September 2017 (0.3 ± 0.1, t_13_ = 3.1 *P* = 0.01, Fig. 3). In addition, E/L ratios were also higher after moult (0.9 ± 0.8) than before laying the following season (0.5 ± 0.3, t_13_ = 3.50, *P* < 0.01, Fig. 4).

**Figure 5:**
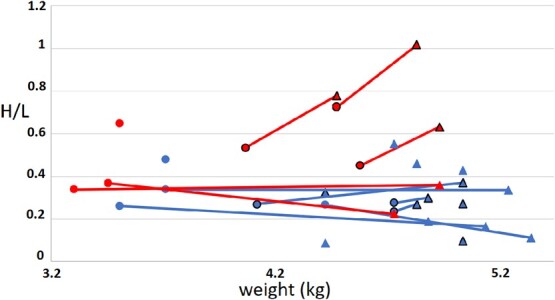
Heterophil/lymphocyte (H/L) vs. weight (in kg) of adult Magellanic penguin during pre-laying (triangles) and chick rearing (circles) in the tourism area (higher H/L values) and no tourism areas (lower H/L values) in 2017 season. Males (with outline) females (without outline). Values for the same individuals joined with lines.

The same individuals belonging to tourism and no tourism areas were sampled during pre-laying and chick rearing in 2017 season. There were no differences between sexes in H/L values of individuals (*n* = 8 females and *n* = 7 males, F_1,14_ = 1.8, *P* = 0.19), therefore both sexes were grouped for the following analysis. There were differences in H/L values between no tourism and tourism areas, being higher in the tourism area than the no tourism area, with no differences between breeding stages (H/L: 1.0 ± 0.7 vs. 0.4 ± 0.2, area: t_12_ = 2.8, *P* = 0.02, stage: t_12_ = 0.7, *P* = 0.48). Individual identity explained 50% of variance. The sampled individuals were also weighed at the beginning and middle of the season. Both males and females lost weight throughout the season with no differences between areas (early vs. middle: F_1,42_ = 16.9, *P* < 0.001; sex: F_1,42_ = 12.0, *P* = 0.001; area: F_1,42_ = 0.5, *P* = 0.46). Females went from an average of 4.5 ± 0.6 kg before egg laying to 3.6 ± 0.4 kg during chick rearing and males went from an average of 4.7 ± 0.5 kg to 4.4 ± 0.2 kg (Fig. 5).

There were significant differences in H/L values between chick rearing 2017 and 2018, with H/L being higher in 2018 than 2017 in both areas 0.5 ± 0.2 in 2017 vs. 1.7 ± 0.6 in 2018 for tourism and 0.3 ± 0.1 in 2017 vs. 0.6 ± 0.1 in 2018 for no tourism (t_10_ = 2.4, *P* = 0.03 and t_6_ = 5.4, *P* = 0.001, respectively). Individual identity explained 45% of variance as there were big individual differences in values (Fig. 5).

### Across the years

#### Adults

**Figure 6:**
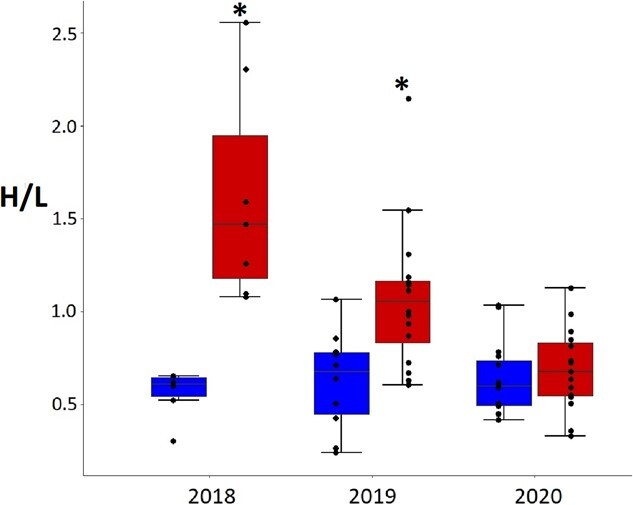
Adult Magellanic penguin heterophil to lymphocyte ratio in tourism (on the right) and no tourism areas (on the left) in 2018 (*n* = 7 and *n* = 6), 2019 (*n* = 16 and *n* = 10) and 2020 seasons (*n* = 15 and *n* = 15). Box plots with * (tourism in 2018 and tourism in 2019) significantly different from all the rest.

During chick rearing, H/L ratios differed amongst areas, years and the interaction of area and year (F_1,63_ = 5.9, *P* = 0.004; F_2,63_ = 36.9, *P* < 0.001 and F_2,63_ = 9.4, *P* < 0.001, respectively). H/L ratios were higher for adults in the tourism area than for the no tourism area in 2018 and 2019 (Table 2, Fig. 6). There were no differences between no tourism and tourism areas in 2020 and with the no tourism areas in 2018 and 2019.

**Table 2:** Two-way comparison *t* statistic and significance (significant differences in bold at *P* < 0.05) between log_10_heterophil vs. lymphocyte ratio for adult Magellanic penguins (*Spheniscus magellanicus*) in areas with (t) and without tourist visitation (n) at Martillo Island in 2018, 2019 and 2020 breeding seasons

**H/L**		**2018**		**2019**		**2020**	
		**n**	**t**		**t**	**n**	**t**
**2018**	**n**		t_11_ = 5.3 ***P* < 0.001**	t_14_ = 0.2 *P* = 0.80	t_20_ = 3.7 ***P* < 0.001**	t_19_ = 0.7 *P* = 0.47	t_19_ = 1.1 *P* = 0.27
	**t**			t_15_ = 5.7 ***P* < 0.001**	t_21_ = 2.6 ***P* = 0.01**	t_20_ = 5.7 ***P* < 0.001**	t_20_ = 5.3 ***P* < 0.001**
**2019**	**n**				t_24_ = 4.1 ***P* < 0.001**	t_23_ = 0.55 *P* = 0.60	t_23_ = 0.99 *P* = 0.33
	**t**					t_29_ = 4.0 ***P* < 0.001**	t_29_ = 3.5 ***P* < 0.001**
**2020**	**n**						t_28_ = 0.5 *P* = 0.62

**Figure 7:**
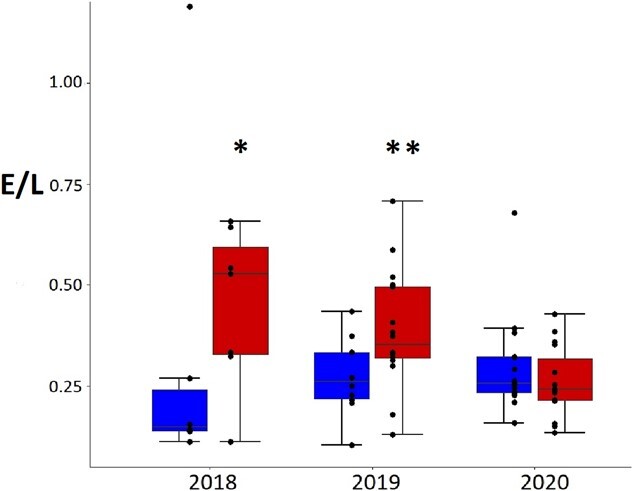
Adult Magellanic penguin eosinophil to lymphocyte ratio in tourism (on the right) and no tourism areas (on the left) in 2018 (*n* = 7 and *n* = 6), 2019 (*n* = 16 and *n* = 10) and 2020 seasons (*n* = 15 and *n* = 15). Box plot with ^*^ (tourism in 2018) different from no tourism in 2018 and tourism in 2020. Box plot with ^**^ (tourism in 2019) different from no tourism in 2018 and tourism in 2020.

E/L ratios differed between areas some years (effect of area alone F_1,63_ = 2.9, *P* = 0.09, effect of year alone F_2,63_ = 0.9, *P* = 0.4, and effect of interaction area and year F_2,63_ = 3.4, *P* = 0.04). In 2018, E/L ratios were higher in the tourism area than the no tourism area. The tourism area in 2020 had lower values than in 2018 and 2019 (Fig. 7, Table 3).

**Table 3:** Two-way comparison *t* statistic and significance (significant differences in bold at *P* < 0.05) between log_10_eosinophil vs. lymphocyte ratio for adult Magellanic penguins (*Spheniscus magellanicus*) in areas with (t) and without tourist visitation (n) at Martillo Island in 2017, 2018, 2019 and 2020 breeding seasons

**E/L**		**2018**		**2019**		**2020**	
	**n**	**t**	**n**	**t**	**n**	**t**	
**2018**	**n**		t_11_ = 2.3 ***P* = 0.03**	t_14_ = 0.7 *P* = 0.49	t_20_ = 2.2 ***P* = 0.03**	t_19_ = 1.1 *P* = 0.27	t_19_ = 0.5 *P* = 0.60
	**t**			t_15_ = 1.8 P = 0.07	t_21_ = 0.4 *P* = 0.67	t_20_ = 1.6 *P* = 0.11	t_20_ = 2.2 ***P* = 0.03**
**2019**	**n**				t_24_ = 1.8 *P* = 0.08	t_23_ = 0.5 *P* = 0.65	t_23_ = 0.3 *P* = 0.80
	**t**					t_29_ = 1.5 *P* = 0.14	t_29_ = 2.3 ***P* = 0.03**
**2020**	**n**						t_28_ = 0.8 *P* = 0.44

#### Chicks

**Figure 8:**
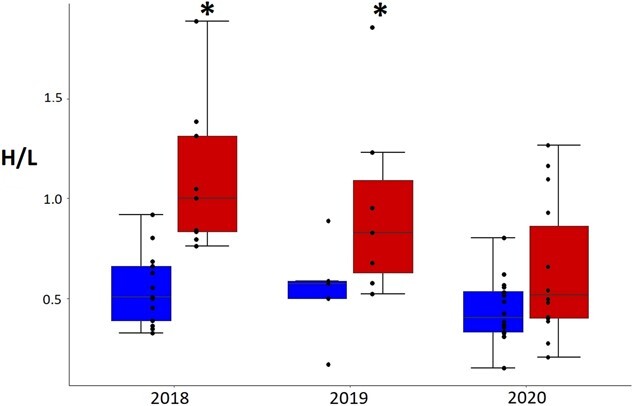
Magellanic penguin chick heterophil to lymphocyte ratio in tourism (on the right) and no tourism areas (on the left) in 2018 (*n* = 9 and *n* = 13), 2019 (*n* = 8 and *n* = 6) and 2020 seasons (*n* = 14 and *n* = 16). Box plots with * (tourism in 2018 and tourism in 2019) different to all the rest except for each other.

**Figure 9:**
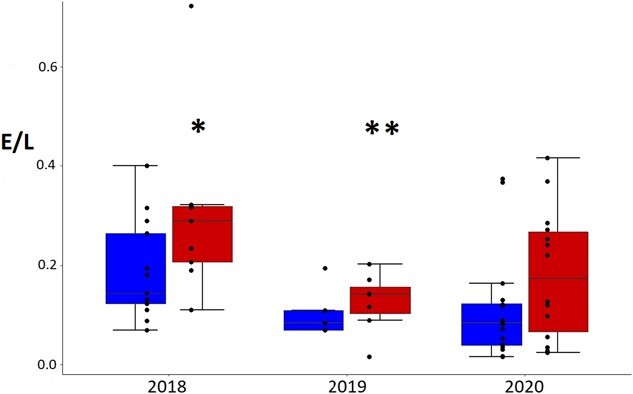
Magellanic penguin chick eosinophil to lymphocyte ratio in tourism (on the right) and no tourism areas (on the left) in 2018 (*n* = 9 and *n* = 13), 2019 (*n* = 8 and *n* = 6) and 2020 seasons (*n* = 14 and *n* = 16). Box plot with * (tourism in 2018) different from no tourism in 2019, no tourism in 2020 and tourism in 2020. Box plot with ** (tourism in 2019) different from no tourism in 2020.

H/L ratios of chicks differed between years and areas (effect of year alone F_2,60_ = 6.1, *P* = 0.004, effect of area alone F_1,60_ = 19.2, *P* < 0.001 and effect of interaction area year F_2,60_ = 1.5, *P* = 0.24). H/L ratios were higher for the tourism area than the no tourism area in 2018 and in 2019 but not in 2020 (Fig. 8). In 2020, H/L values were lower and not different from the no tourism area (Table 4). These results are equivalent to adults. For E/L ratios, values differed only amongst years, but not areas or the interaction of area and year (effect of year F_2,60_ = 6.7, *P* = 0.002, effect of area alone F_1,60_ = 3.2, *P* = 0.07 and effect of interaction area year F_2,60_ = 0.4, *P* = 0.67). E/L values were only significantly higher in the tourism area in 2018 (Fig. 9). No differences were apparent between areas in other years (Table 5).

**Table 4:** Two-way comparison *t* statistic and significance (significant differences in bold at *P* < 0.05) between log_10_ heterophil vs. lymphocyte ratio for Magellanic penguin (*Spheniscus magellanicus*) chicks in areas with (t) and without tourist visitation (n) at Martillo Island in 2018, 2019 and 2020 breeding seasons

**H/L**		**2018**		**2019**		**2020**	
		**n**	**t**	**n**	**t**	**n**	**t**
**2018**	**n**		t_20_ = 3.7 *P* **< 0.001**	t_17_ = 0.4 *P* = 0.72	t_19_ = 2.5 ***P* = 0.02**	t_27_ = 1.4 *P* = 0.16	t_27_ = 0.3*P* = 0.74
	**t**			t_13_ = 3.2***P* = 0.002**	t_15_ = 0.9 *P* = 0.39	t_23_ = 5.2***P* < 0.001**	t_21_ = 3.5***P* < 0.001**
**2019**	**n**				t_12_ = 2.3 ***P* = 0.02**	t_20_ = 0.7 *P* = 0.50	t_18_ = 0.6*P* = 0.54
	**t**					t_22_ = 3.8 ***P* < 0.001**	t_20_ = 2.3***P* = 0.03**
**2020**	**n**						t_28_ = 1.8*P* = 0.07

**Table 5:** Two-way comparison *t* statistic and significance (significant differences in bold at *P* < 0.05) between log_10_ eosinophyl vs. lymphocyte ratio for Magellanic penguin (*Spheniscus magellanicus*) chicks in areas with (t) and without tourist visitation (n) at Martillo Island in 2018, 2019 and 2020 breeding seasons

**E/L**		**2018**		**2019**		**2020**	
	**n**	**t**	**n**	**t**	**n**	**t**	
**2018**	**n**		t_20_ = 1.4 *P* = 0.17	t_17_ = 1.3 *P* = 0.20	t_19_ = 1.3 *P* = 0.2	t_27_ = 2.7 ***P* = 0.01**	t_27_ = 0.9 *P* = 0.40
	**t**			t_13_ = 2.3 ***P* = 0.02**	t_15_ = 2.4 ***P* = 0.02**	t_23_ = 3.8 ***P* < 0.001**	t_21_ = 2.2 ***P* = 0.03**
**2019**	**n**				t_12_ = 0.1 *P* = 0.91	t_20_ = 0.6 *P* = 0.54	t_18_ = 0.7 *P* = 0.50
						t_22_ = 0.8 *P* = 0.40	t_20_ = 0.6 *P* = 0.54
**2020**	**n**						t_28_ = 1.8 *P* = 0.07

### Trophic values

**Figure 10:**
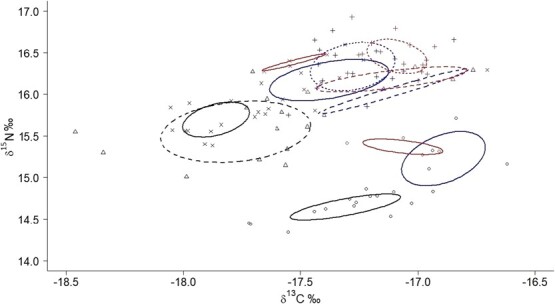
Biplot of blood δ^13^C and δ^15^N and SEAc of adult Magellanic penguins in areas visited by tourists (red) and not visited by tourists (blue) and chicks in area not visited by tourists (black). 2017 (circles and SEAc with filled line), 2018 (triangles and SEAc with dashed line), 2019 (crosses and SEAc with pointed line) and 2020 (exes and SEAc with filled line).

Isotopic values in blood of adult chick rearing penguins differed amongst years (F_3,62_ = 9.6, *P* < 0.001 for δ^13^C and F_3,62_ = 14.7, *P* < 0.001 δ^15^N). Particularly in 2017 δ^13^C was less negative than 2019 and 2020, and δ^15^N was inferior in 2017 than the remaining years (Table 6). No differences were detected between areas with and without tourism for δ^15^N (F_1,62_ = 0.2, *P* = 0.69) and only marginally for δ^13^C (F_1,62_ = 3.8, *P* = 0.05, *n* = 63). Chicks had more negative δ^13^C and lower δ^15^N than adults within each year (in 2017: F_1,21_ = 7.6, *P* = 0.004 and F_1,21_ = 30.1, *P* < 0.001; in 2018: F_1,18_ = 4.7, *P* = 0.02 and F_1,18_ = 4.8, *P* = 0.02, and in 2020: F_1,26_ = 17.7, *P* < 0.001 and F_1,26_ = 20.8, *P* < 0.001). In consonance with adults, isotopic values for chicks were more enriched in 2020 and 2018 in contrast with 2017 (t_37_ = 4.63, *P* < 0.001 and t_37_ = 3.77, *P* < 0.001 for δ^13^C and t_37_ = 5.82, *P* < 0.001 and t_37_ = 8.16, *P* < 0.001 for δ^15^N, Fig. 10).

**Table 6:** δ^13^Carbon (corrected for the Suess effect) and δ^15^Nitrogen values for adult Magellanic penguins at Martillo Island in 2017, 2018, 2019 and 2020 in areas with (t) and without tourism (n)

	**2017**		**2018**		**2019**		**2020**	
**δ** ^**13**^ **C**	**n**	**t**	**n**	**t**	**n**	**t**	**n**	**t**
	−16.9 ± 0.2	−17.1 ± 0.2	−17.1 ± 0.3	−17.1 ± 0.3	−17.3 ± 0.2	−17.1 ± 0.2	−17.5 ± 0.4	−17.5 ± 0.2
			2017 vs. 2018 t = 1.1 *P* = 0.27		2017 vs. 2019 t = 2.5 ***P* = 0.01**		2017 vs. 2020 t = 5.0 ***P* < 0.001**	
**δ** ^**15**^ **N**	15.2 ± 0.3	15.3 ± 0.1	16.0 ± 0.3	16.2 ± 0.2	16.3 ± 0.3	16.3 ± 0.7	16.1 ± 0.3	16.4 ± 0.1
			2017 vs. 2018 t = 3.8 ***P* < 0.001**		2017 vs. 2019 t = 6.6 ***P* < 0.001**		2017 vs. 2020 t = 5.0 ***P* < 0.001**	

The SIBER ellipse area overlap as a proportion of non-overlapping areas amongst years was lowest in 2017 compared with the remaining years: 2017–2018 = 7%, 2017–2019 = 0%, 2017–2020 = 0%, 2018–2019 = 24%, 2018–2020 = 46%, 2019–2020 = 33%. In 2017 the overlap between chicks (SEAc = 0.08) and adults in no tourism area (SEAc = 0.22) was 18% and with the adult tourism area (SEAc = 0.06) was 0%, tourism vs. no tourism area was 20% overlap. In 2018, ellipse area overlaps between chicks (SEAc = 0.40) and adults in no tourism area (SEAc = 0.11) was 14%, with adults in the tourism area (SEAc = 0.21) was 22% and adults in tourism vs. no tourism areas was 27% overlap. In 2019, overlap between adults in no tourism (SEAc = 0.17) and tourism area (SEAc = 0.08) was 43%. In 2020, overlap between chicks (SEAc = 0.09) and adults in no tourism area (SEAc = 0.20) was 21%, between chicks and adults in tourism (SEAc = 0.02) was 4% and adults in no tourism vs. tourism was 11%. Within each year, tourism and no tourism adults had a 10–45% overlap and chicks had a 0–20% overlap with adults.

### Natural and anthropogenic factors in 2017–2020

**Figure 11:**
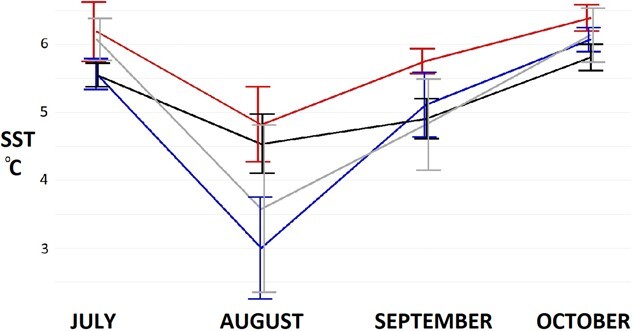
Monthly average SST ± SD for the area surrounding Martillo Island in 2017 (mean and range shown on the left for each month), 2018 (second from the left), 2019 (third from the left) and 2020 (mean and range shown on the right for each month).

Average monthly sea surface temperatures (SST) differed amongst months, years and their interaction (F_11,44_ = 435, *P* < 0.001; F_3,44_ = 10, *P* < 0.001 and F_33,44_ = 4, *P*< 0.001, respectively). Average SST were higher for the area surrounding the colony in the winter of 2017 (Fig. 11), particularly in September in comparison with the same month in the remaining years (5.8 ± 0.2°C vs. 5.1 ± 0.5°C in 2018: t = 2.5, *P* = 0.02; vs. 4.9 ± 0.3°C in 2019: t = 3.3, *P* = 0.003; vs. 4.8 ± 0.7°C in 2020: t = 3.6, *P* = 0.002).

Rainfall differed amongst years and the interaction of month and year but not amongst months alone (F_3,44_ = 8, p < 0.001, F_33,44_ = 2, *P* = 0.006 and F_11,44_ = 1, *P* = 0.52, respectively). Average rainfall was higher in November 2017 (4.5 mm) than the rest of the years: 1.6 mm in 2018 (t = 3.4, *P* < 0.001), 1.9 mm in 2019 (t = 3.4, *P* < 0.001) and 0.5 mm in 2020 (t = 5.3, *P* < 0.001). Average and minimum ambient temperature differed amongst months, years and their interaction (by month: F_11,44_ = 156, *P* < 0.001 and F_11,44_ = 146, *P* < 0.001; year: F_3,44_ = 6, *P* < 0.001 and F_3,44_ = 5, *P* = 0.002; and interaction: F_33,44_ = 3, *P* < 0.001 and F_33,44_ = 3, *P* < 0.001). In addition, average (8.9 ± 2°C) and minimum ambient temperatures (4.1 ± 2°C) were lower in December 2017 than the remaining years: in 2018 = 10.0 ± 2°C and 5.4 ± 2°C (t = 1.94, *P* = 0.05 and t = 2.50, *P* = 0.01), 10.0 ± 2°C and 5.4 ± 2°C for 2019 (t = 1.91, *P* = 0.05 and t = 2.39, *P* = 0.02) and 9.5 ± 3°C and 5.4 ± 2°C for 2020 (t = 1.00, *P* = 0.31 and t = 2.49, *P* = 0.003). Therefore, 2017 had wetter and cooler conditions during breeding than the remaining seasons.

**Figure 12:**
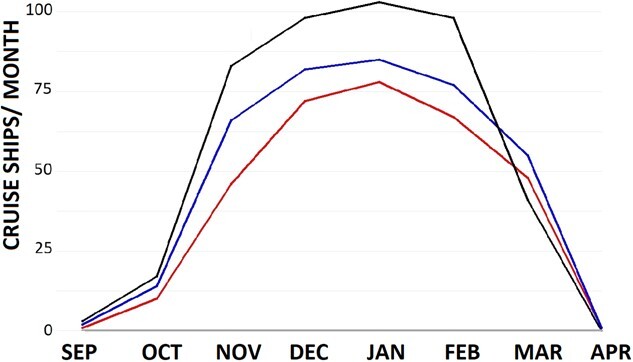
Number of cruise ships entering Ushuaia port per month in 2017 (second lowest values in January), 2018 (second highest values in January), 2019 (highest values in January) and 2020 (no cruise ships).

Marine traffic within the Beagle Channel is composed of a variety of marine vessels, tankers, fishing vessels, cargo ships and particularly in summer, cruise ships become very frequent, except for 2020 when cruise tourism was 0 as a result of the Covid-19 pandemic (Fig. 12). Total marine traffic per year was lower in 2020 (369 vessels docking in Ushuaia between January and December) and 2021 (200 vessels) than the previous years (469 in 2017, 519 in 2018, 533 in 2019). Tourism at Martillo Island usually reaches 6–7 groups of 20 tourists landing at the island each day during the penguin breeding season. These landings reduced to 0 between March and December 2020 and only did tourists begin to return to the Martillo Island colony in January 2021 with a much lower frequency (only once or twice a week) until the end of the season. Therefore, both vessel transit, particularly of cruise ships, and human presence at Martillo Island was lower in 2020 than previous seasons.

## Discussion

Stress levels of Magellanic penguins at Martillo Island, measured by the H/L ratio, differed amongst moments within the season, breeding locations and years. Stress levels while breeding were consistently higher in the tourism area than the no tourism area and this difference was maintained between breeding seasons. Within the breeding season, stress levels were maintained from pre-laying to chick rearing, yet weight of all individuals diminished presumably due to the energetic costs of food provisioning to the growing offspring (Green *et al.,* 2009). Amongst breeding seasons, moulting seemed to generate higher stress than pre-laying the following season. In addition, trophic levels also varied amongst seasons and stress levels while breeding were also higher in a season when trophic levels were also higher than in the previous season with lower stress and trophic levels. In the 2020 season, stress levels of individuals in the tourism area were similar to the no tourism area both that season and in previous seasons, coinciding with the reduction of tourist visitation and marine traffic due to the Covid-19 pandemic.

### Individual changes

Enhanced stress while breeding may be due to external factors such as changes in prey availability, extreme weather conditions, competition for breeding grounds, predators, human presence, or internal factors such as nutritional stress, poor body condition, prevalence of endo or ectoparasites, among others (Gandini *et al.,* 1994; Fowler *et al.,* 1995; Frere *et al.,* 1998; Walker *et al.,* 2005b; Brandāo *et al.,* 2011; Boersma and Rebstock, 2014). A combination of factors is likely at play at a given time. During breeding, individuals are driven to a state of enhanced energetic demands while constrained in foraging time to successfully fulfil parental duties (Furness, 1978; Sala *et al.,* 2015). In this state it is expected individuals will reach higher stress levels triggered by one or several of the before mentioned factors.

Penguins are long-lived seabirds, and once they reach adulthood are able to breed annually throughout their lifetime. At the beginning of the breeding season individuals must reach a threshold of nutritional and physiological conditions in order to withstand the breeding season as they must invest in breeding as well as self-maintenance (Yorio and Boersma, 1994; Kulaszewics *et al.,* 2016; Rebstock and Boersma, 2018). This energetic demand was detected in the weight changes individuals, and particularly females, go through within the season, losing about 1 kg between pre-laying and chick rearing in the case of females. Stress levels did not differ from pre-laying to chick rearing yet their body condition deteriorated as they lost weight (Fowler *et al.,* 1994). In addition, stress levels were higher at the end of the season during pre-moult, than at the beginning of the following season, most likely linked with the energy investment associated with feather production and the need for fasting until new feathers grow in and they can return to sea (Cherel *et al.,* 1994). While wintering, individuals reduce their stress levels before they begin a new breeding event.

Trophic levels are also important when assessing individual stress while breeding (Benowitz-Fredericks *et al.,* 2008; Will *et al.,* 2015). In 2017, individuals had lower stress levels and trophic levels than the following season when they were resampled. This trophic shift may be due to a diet shift as individuals are likely to have fed on a higher proportion of lower trophic level squat lobster (*Munida gregaria*) than higher level Fuegian sprat (*Sprattus fuegensis*) in the former year and increased the proportion of Fuegian sprat the following season. Squat lobster may be easier to obtain as individuals remain suspended in the water column and do not attempt to flee like Fuegian sprat (see video footage in Harris *et al.,* 2023), and therefore stress levels modulated by foraging effort of breeding individuals was lower that season. It is important to keep in mind that all sampled individuals in the current study were able to raise at least one chick to fledging, therefore endured the challenges of the given breeding season successfully. The sampling of unsuccessful breeders in further studies will help fill the gap regarding the conditions in which a breeding event is abandoned. Less experienced individuals may be less prone to endure stressful conditions and as breeding events go b,y individuals may adjust to the breeding conditions, improve their mechanisms to detect and hone in on prey, forage more efficiently, improve their timing of arrival at the colony, and even become habituated to human presence. As they age, their body condition may eventually begin to deteriorate as they reach senescence (Wasser and Shernam, 2010). When the prospect of future breeding events becomes dimmer, individuals may invest more and accept a higher cost in order to follow through with a breeding event (i.e. *Uria aalge*, Reed *et al.,* 2008; Froy *et al.,* 2013). Stress levels of older breeding individuals under equal conditions may be higher than younger breeding individuals (as occurs with wandering albatross *Diomedea exulans*, Angelier *et al.,* 2006). Individual differences are also important particularly in species with high variability and behavioural plasticity such as the Magellanic penguin (Boersma *et al.,* 1990; Sala *et al.,* 2014). In the current study, variability in stress levels amongst individuals accounted for up to 50% of total variability in the data. Therefore, long-term data on the same identified individuals will shed light on other aspects of their behaviour and physiology that may be masked in one-time data collecting events. Future research will take into account the age and identity of breeding individuals in order to factor it into the breeding decisions they make and their performance.

### Tourism and no tourism areas

Magellanic penguins breeding in the tourism area at Martillo Island had higher stress levels than in the no tourism area most seasons. These findings are in line with those at other colonies with tourist visitations which had higher stress levels than individuals not exposed to tourists, particularly at Punta Tombo (Fowler, 1999; Walker *et al.,* 2008; Palacios *et al.,* 2018). E/L values were also higher in the tourism exposed area than the no tourism area, which could be linked to a higher prevalence of endoparasites in the tourism area (such as gastrointestinal helminths, Diaz *et al.,* 2010). Human presence alone may be elevating basal stress responses in the penguins breeding within the tourist trail and the presence of endo or ectoparasites may be due either to higher infection rate in this area or a weaker immunity of individuals rendering them unable to fight off infections (Esparza *et al.,* 2004; Owen *et al.,* 2010). However, other factors may be at play such as microclimatic conditions, nest density, etc, which may differ between zones (Rivera-Parra *et al.,* 2014; Ramos and Drummond, 2016).

At Martillo Island, the area surrounding the tourist trail has loose soil and gravel with low vegetation cover. This kind of substrate seems to correlate with a higher presence of fleas (personal observation). The erosion generated by penguins and introduced muskrat (*Ondatra zibethicus*) in the areas of older occupation may be contributing to the presence of fleas and possibly other parasites in the nesting grounds. The no tourism area was colonized by penguins at roughly the same time as the tourist visited area but the terrain characteristics and elevation make it a different nesting environment. Nests are covered by vegetation and soil is more compact (Quiroga *et al.*, 2020). The soil surrounding the nests is moist, which may in turn reduce the presence of fleas and larvae in the ground (Waller *et al.,* 2020). Ectoparasite prevalence has even been reported to be the cause of desertion of a breeding seabird, the Guanay cormorant (*Phalacrocorax bougainvilli,*Duffy, 1983). The presence of ectoparasites is directly linked to enhanced heterophil counts, which may also increase the H/L ratio and also inflict higher stress levels in individuals coping with the fleas feeding on them (Lehmann, 1993; Al-Mawla and Al-Saffar, 2008). In addition, environmental conditions at the nest may be regulating the prevalence of endo and ectoparasites as cooler, wetter seasons generate poorer conditions for larva and flea survival (Hebb *et al.,* 2000; Espinaze *et al.,* 2020; Waller *et al.,* 2020). Future research should also focus on *in situ* measurements of temperature and humidity to have more precise information on microclimatic conditions.

Higher stress may also be due to increased defence of more desirable nesting sites. This density dependent stress response has been observed in other penguin species (Viblanc *et al.,* 2012, 2014). Fights over nesting sites tend to occur early in the season and males that win the fights are then rewarded with nest sites that a female will likely approve (Renison *et al.,* 2002). The tourism area has one of the highest nest densities and therefore competition amongst neighbours for nesting sites and a high production of future prospecting candidates for nesting in that area, considering the philopatric behaviour of penguins. Given that nest density is at its limit in some sectors, that nests may cave in and digging of new caves is energetically demanding, the number of nesting sites tends to remain constant or even decrease over time, therefore conflict over these locations is expected to increase (Scioscia *et al.* in preparation). A percentage of non-breeders are often seen ambling amongst the nests or even occupying empty nests in this area (personal observation). This area is adjacent to the eastern facing sector of the beach that becomes most crowded with penguins throughout the season, and increasingly more so after the arrival of juveniles in January. Ectoparasite transmission may also be higher when penguin densities are higher (Espinaze *et al.,* 2019). The no tourism area also has a high density of nests in some sectors, yet vegetation and terrain inclination fragment the area into smaller sectors with nests. Given that nests are dug out in firmer soil, nest caving is less common and therefore, nest owners may become more permanent over time (Stokes and Boersma, 1991). In addition, the no tourism area is less accessible from the beach and is therefore not recurrently occupied by non-breeders (personal observation). Stress levels of breeders in this area may be lower due to these differences in location and behaviour of individuals in comparison with the tourist area.

### Trophic values in contrasting years

Stress levels were lower for the same individuals in 2017 than 2018 in both areas, and a trophic shift was also detected in individuals, indicating some change in prey may have occurred that year. Trophic level of chicks mirrored that of adults, as in 2017 levels were lower than the remaining years. In 2017, sea surface temperature surrounding the colony was higher during the productive phase of the annual cycle (September–October) before water stratification intensifies and separates the organic and the light-receiving phases of the water column (Flores Melo *et al.,* 2020). This may have increased the productivity, causing a shift in the trophic value of the primary feeders or even the abundance and distribution of the secondary feeders (squat lobster and Fuegian sprat) which in turn changed the trophic value of top predators such as penguins in this particular system (Riccialdelli *et al.*, 2020). Diet composition may have changed with individuals feeding on a lower trophic level and more predictable food source (such as pelagic squat lobster) than the less predictable higher trophic level Fuegian sprat (Riccialdelli *et al.*, 2020). The presence of Fuegian sprat in the area penguins feed depends on the oceanographical regime at the moment penguins were feeding (as suggested in Scioscia *et al.,* 2014; Diez *et al.,* 2018). In 2018, 2019 and 2020, trophic levels were similar amongst individuals independently from their breeding location. Trophic shifts seem to have an important print on physiology as body condition is intimately linked to diet composition (Wanless *et al.,* 2005; Wilson *et al.,* 2005; Jodice *et al.,* 2006; Barrionuevo *et al.,* 2018; Colominas-Ciuró *et al.,* 2022b). Changes in diet correlating to changes in stress levels may indicate slightly better or worse breeding seasons even if these changes do not visibly impact on breeding success (Welcker *et al.,* 2009; Blanco *et al.,* 2022). These diet shifts, as have been detected in the past (Scioscia *et al.,* 2014), may influence the stress levels of individuals in a particular season (Barger and Kitaysky, 2011; Dunphy *et al.,* 2020). Individuals that endure higher stress during suboptimal years may reduce their breeding probability the following season or ultimately trigger nest desertion at a given moment of the season (Boersma *et al.,* 1990).

Environmental variables such as rainfall also have an impact on Magellanic penguin breeding events, particularly during early chick rearing (Boersma and Rebstock, 2014). In addition, wetter seasons may have a positive impact by reducing endo and ectoparasite prevalence at the nesting site, which in turn is expected to reduce stress levels of individuals (Waller *et al.,* 2020). Ambient temperature is also important as heat may be an important factor in sublethal increases in stress levels triggered by elevated temperatures and even mortality due to heat stress has been recorded in other colonies (Holt and Boersma, 2022). In the 2017 season, rainfall was higher during incubation-early chick rearing (November), and chick rearing (December) was cooler, which may have reduced the incidence of ectoparasites that year (Espinaze *et al.,* 2020), which in turn may have lowered H/L values. In 2018–2020, environmental conditions were similar and stress levels were also similar, particularly in the no tourism area. Long-term data sets covering different environmental conditions will help understand the direct and/or indirect impacts of climate on penguin stress levels in time.

### What happened in 2020?

In 2020 there was hardly any tourism, landing of visitors on Martillo Island started in January 2021 and only twice a week. Vessel transit in the Beagle Channel was also greatly reduced given the Covid-19 pandemic situation. Tourist vessel transit within the Channel is usually particularly high during the penguin breeding season, with 300–450 cruise ships transiting the Channel between September and March, except for 2020 when cruise ship traffic was 0. Movement of vessels may commonly condition the distribution of the marine wildlife (Bas *et al.,* 2017; Sprogis *et al.,* 2020) which may have reversed in the absence of vessel traffic during the pandemic. In 2020, schools of Fuegian sprat may have remained more persistent in sectors of the channel commonly disturbed by passing vessels, and easier to detect by the penguins. Stress levels in the tourism area were like the no tourism area, and equivalent results were obtained for chicks. Semi continuous human presence *per se* may also be increasing basal stress levels of chicks and penguins breeding in proximity of the tourist trail. Chicks may be more prone to suffer from stress due to lack of habituation to a potential predator (Ellenberg *et al.,* 2009). Elevated stress levels have also been detected in chicks from tourist visited areas in another Magellanic penguin colony (Walker *et al.* 2005a). Adults, on the other hand, may become habituated to human presence over successive breeding events, reducing their fight or flight response (Cevasco *et al.,* 2001; Walker *et al.,* 2006; Villanueva *et al.,* 2011). In areas of the colony with less human presence, behaviours such as head turning, biting and even fleeing is more commonly observed than close to the tourist trail (personal observation, equivalent to Yorio and Boersma, 1992). The lack of ‘stressed’ behaviour may not imply individuals are not stressed as they may endure long-term physiological stress triggered by chronically elevated basal glucocorticoids, which in turn hampers their immune system, making them more prone to pathogens (Koutsos and Klasing, 2014). Within the tourist-visited area, other slighter effects of human presence such as increased movement of the penguins both within or amongst the nests may encourage ectoparasite transmission from one penguin to another (personal observation). A combination of disturbed marine environment, direct human presence and ectoparasite prevalence may be generating the observed effects on penguin physiology.

## Conclusions

Stress of Magellanic penguins is likely influenced by breeding status, location and natural and anthropogenic factors. Particularly in the 2020 season, stress levels of adults and chicks in the tourism area were lower than other years with similar natural conditions. Future research should include multiple approaches: diet, stress, age, behaviour, demography, parasite prevalence, etc, to better understand and describe what may be influencing penguin physiology, behaviour and breeding performance each year. In a globalized world where anthropic effects can no longer be eliminated completely, the monitoring of wildlife populations integrated in a human-impacted environment is key to ensure the conservation of these iconic species over time.

## Data Availability

The datasets generated during the current study are available from the corresponding author on request.
